# From Cardiac Mystery to Dental Discovery: Resolving Recurrent Infection in a Prosthetic Heart Valve Patient

**DOI:** 10.7759/cureus.40073

**Published:** 2023-06-07

**Authors:** Elias Nabhan, Marie-Michelle Kawas, Rana Tohme, Samer R Nasr

**Affiliations:** 1 Cardiology Division, University of Balamand, Beirut, LBN; 2 General Medicine, University of Balamand, Beirut, LBN; 3 Cardiology Division, Mount Lebanon University Hospital, Beirut, LBN; 4 Head of Cardiology Division, University of Balamand, Beirut, LBN

**Keywords:** abscess, bacteremia, dental abscess, endocarditis, prosthetic valve

## Abstract

We present the case of a 71-year-old female with a history of surgical bioprosthetic aortic valve replacement who developed a liquefactive abscess near the mitral valve trigone following Streptococcus gallolyticus bacteremia. The patient initially presented with dyspnea and symptoms of an upper respiratory tract infection. A trans-esophageal echocardiogram revealed mitral valve vegetation and a possible source of sepsis near the prosthetic aortic valve. However, it was the identification of multiple silent dental abscesses during a routine dental check-up that led to the resolution of the patient's symptoms and the eradication of the infectious process. This case highlights the importance of considering dental infections as a potential cause of recurrent bacteremia and infectious complications in patients with prosthetic heart valves.

## Introduction

Infective endocarditis is a serious condition that affects the inner lining of the heart chambers and valves [1]. It occurs when bacteria, fungi, or other microorganisms enter the bloodstream and attach to damaged areas of the endocardium, leading to the formation of vegetation. Common causative agents include bacteria such as Staphylococcus aureus, Streptococcus viridans, and Enterococcus species, as well as fungi like Candida species [2]. Risk factors for infective endocarditis include mainly prior heart valve surgery, congenital heart defects, and intravenous drug use [3].

Prosthetic heart valves are particularly vulnerable to infective endocarditis compared to native heart valves due to their potential for bacterial attachment and biofilm formation, making treatment challenging as antibiotics struggle to penetrate these biofilms. Infective endocarditis can result in severe complications, including periannular extension of infection, abscess formation, valvular dysfunction, dehiscence, rupture, or fistula [4]. Abscess formation is more commonly associated with aortic valve infective endocarditis, especially in cases involving prosthetic valves. In such situations, surgical intervention can be a potential treatment option, particularly when accompanied by heart failure. Trans-esophageal echocardiogram (TEE) is the modality of choice for the initial assessment of any patient at risk for perivalvular extension of infective endocarditis. An echo-free space suggests that complete liquefaction of the affected cardiac tissue has occurred [5].

Periannular extension of infection or abscess formation is considered one of the most serious complications of infective endocarditis, often necessitating surgical therapy for eradication of the infection, drainage of abscess cavities, excision of necrotic tissue, closure of fistulous tracts, and possibly valve-replacement surgery. However, delayed decision-making regarding the surgery can make valve replacement more challenging, especially when extensive destruction of periannular supporting tissues ensues [6].

We present the case of a patient with a history of prosthetic aortic valve replacement and Streptococcus gallolyticus bacteremia. The timely identification and management of the infection source led to symptom resolution and the eradication of the infectious process.

## Case presentation

A 71-year-old female non-smoker and non-alcoholic known to have hypertension, paroxysmal atrial fibrillation, and a history of surgical bioprosthetic aortic valve replacement seven months ago presented with chills, dyspnea, a productive cough, and fatigue of a few days duration. On admission, her temperature was 39.3 C, her blood pressure was 141/74 mmHg, and her pulse was 87 bpm. A physical exam showed left basal rhonchi that were otherwise unremarkable. The review of the systems is clear.

Her last dental check-up was two weeks before valve replacement, and notably, she has good oral hygiene with no recent dental manipulations or surgeries.

Her ECG showed a sinus rhythm without any significant abnormalities. Her pertinent laboratory results are shown in Table 1.

**Table 1:** Pertinent laboratory findings WBC: white blood cells; LDH: lactate dehydrogenase; CRP: C-reactive protein

Test name	Unit	Current result	Normal range
WBC	10³/ul	15.06	4-11
Neutrophils	%	87.9	40-70
Lymphocytes	%	13.2	20-45
Haemoglobin	g/dl	10.1	12-15.5 in females
LDH	IU/L	332	<225
CRP	mg/l	254	0.0-6

**Figure 1:**
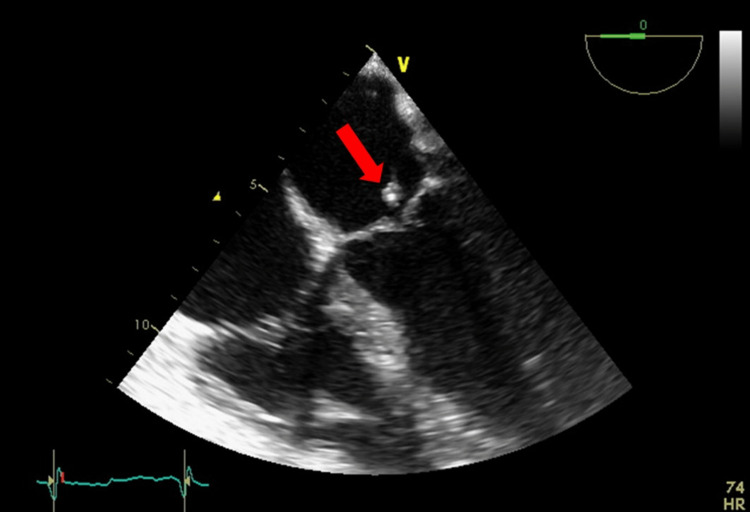
Four chamber view on trans-esophageal echocardiogram (TEE) showing mitral valve vegetation (red arrow). TEE: trans-esophageal echocardiogram

Chest radiography showed minimal left lower lobe infiltrates. Sputum culture showed moderate growth of normal flora. Urinalysis and culture were negative, and blood culture showed growth of Streptococcus gallolyticus after three days of incubation. A TEE was ordered to rule out endocarditis and showed a 12 mm mitral valve vegetation with moderate mitral regurgitation and an intact prosthetic aortic valve (Figure 1). The patient was hence started on intravenous (IV) antibiotics (vancomycin, ampicillin, and ceftriaxone) for six weeks.

**Figure 2:**
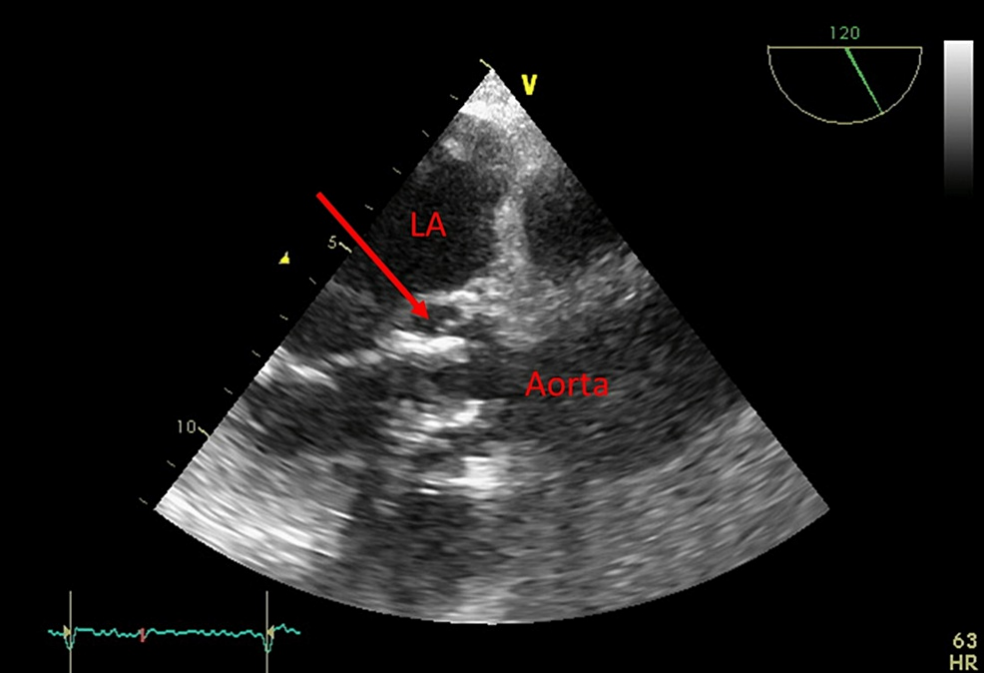
Apical long-axis view on trans-esophageal echocardiogram (TEE) showing a liquefactive abscess (red arrow) near the mitral trigone. LA: left atria; TEE: trans-esophageal echocardiogram

Despite appropriate antibiotic therapy, the patient's improvement was only transient, and she still complains of repetitive episodes of fever reaching 38.5 C and fatigue. Repeated blood cultures showed persistence of Streptococcus gallolyticus bacteremia, and a follow-up TEE showed a quasi-total resolution of the mitral valve vegetation but a new liquefactive abscess near the mitral trigone without a shunt with the left atria (Figure 2).

A positron emission tomography (PET) scan showed an increased uptake near the prosthetic aortic valve, evoking a possible source of sepsis, which could explain the recurrence of the patient’s symptoms. Surgery to replace the prosthetic aortic valve to control the source of infection was discussed with the family; however, the patient refused. A few days later, after visiting her dentist for her routine check-up, she was incidentally found to have multiple asymptomatic dental abscesses despite having good oral hygiene. Extraction of the culprit teeth along with antibiotic therapy (amoxicillin/clavulanic acid) was followed.

Subsequently, the patient experienced significant improvement in her symptoms, and multiple TEEs conducted afterward confirmed the absence of any growths or abscesses in the heart, indicating that the infection had been successfully resolved.

## Discussion

For infectious endocarditis to be established, it requires the trifecta of endocardial damage, exposure to bacteria, and lastly, an organism with appropriate virulence factors. The endocardial injury will be the site of activation of the coagulation cascade and platelets, which leads to the development of sterile vegetation [7]. Those vegetations then require exposure to bacteremia with appropriate virulence to be colonized and infected [8]. Infectious pathogens can originate from any source, including IV drug use, distant infections in the body, dental infections, or manipulations [3].

The majority of infective endocarditis cases are caused by gram-positive bacteria, specifically streptococci, staphylococci, and enterococci. These three groups account for approximately 80% of all cases, with Staphylococcus aureus alone responsible for around 30% of cases in developed countries. While various species of streptococci are common culprits, other bacteria that commonly reside in the oropharynx, such as the Haemophilus, Actinobacillus, Cardiobacterium, Eikenella, and Kingella (HACEK) organisms, can also be responsible, albeit less frequently. Fungal endocarditis is rare, comprising approximately 1% of cases, but it can be a fatal complication, particularly in immunocompromised individuals with systemic Candida and Aspergillus infections [5,1]. Although Streptococcus gallolyticus is an uncommon causative agent of infective endocarditis, it has been associated with a higher prevalence of underlying gastrointestinal malignancies [7,9]. In this case, the initial presentation with respiratory symptoms and subsequent identification of mitral valve vegetation led to the suspicion of endocarditis. However, the resolution of the vegetation and the development of a liquefactive abscess near the mitral valve trigone raised concerns about a persistent source of infection.

The occurrence of prosthetic valve endocarditis (PVE) is most frequent in the initial weeks and months following valve implantation, with a cumulative incidence reaching approximately 3% within one year and 6% within five years [10]. In comparison with native valve endocarditis, prosthetic valve endocarditis is significantly more aggressive and associated with peri-annular infection, tissue invasion, abscess formation, and fistulous perforation [11]. During the active phase of infection, when intravenous antibiotics are administered, surgical intervention is considered a class I indication in specific situations. These indications include the presence of heart failure, the formation of abscesses, valve dehiscence, progressive valve dysfunction, and infection caused by a microorganism that is resistant to treatment or difficult to eradicate [12].

The modified Duke criteria are used to make the diagnosis of infective endocarditis. The criteria include major and minor criteria, and a diagnosis of infective endocarditis is made if there is evidence of either two major criteria, one major and three minor criteria, or five minor criteria. The major criteria consist of positive blood cultures with a common pathogen grown in two separate cultures, positive Coxiella serology, or evidence of endocardial involvement, such as the presence of a new valvular regurgitation murmur or positive echocardiogram results. On the other hand, the minor criteria include the presence of predisposing factors, a fever above 38 C, evidence of vascular or immune phenomena, or microbiologic findings that do not meet the major criteria [12].

Nonetheless, diagnosis of infective endocarditis is challenging, especially when it is not associated with vegetations or when the degenerative changes of prosthetic valves can be falsely thought to be vegetations [13,14]. In addition, a blood culture might be falsely negative for organisms that require special culture or if the patient has been receiving antibiotics [15]. Conversely, our patient exhibited persistent vegetation and abscess formation despite receiving a one-month course of antibiotic treatment. This raised the possibility of a persistent infectious source, which was suspected to originate from the aortic prosthetic valve. However, further investigation revealed that the source of the infection was dental in origin, and Streptococcus gallolyticus, found in the oropharyngeal flora, led to dental abscesses and bacteremia, followed by the deposition of vegetation on the mitral valve.

## Conclusions

Dentists should be vigilant and thorough in assessing patients with prosthetic heart valves for possible oral infections. Even in the absence of overt dental symptoms, dental infections should be suspected as a potential source of recurrent bacteremia, especially in patients with prosthetic heart valves.

Collaborative efforts between dental and medical professionals are essential in managing patients with prosthetic heart valves, as identifying and treating dental abscesses at an early stage can effectively resolve the infection and prevent further complications.
